# Supplementary material for: Luminescence Properties of Green Phosphor Ca2Ga2(Ge1-xSix)O7:y%Eu2+ and Application
Source: Materials (Basel). 2023 May 11;16(10):3671. doi: 10.3390/ma16103671 (PMC10223489; doi:10.3390/ma16103671)
Supplement: Supplementary file 1 [file materials-16-03671-s001.zip › materials-2361482-supplementary.pdf]

Supplementary Materials

Luminescence Properties of Green Phosphor $Ca_2Ga_2(Ge_{1-x}Si_x)O_7$:y%$Eu^{2+}$ and Application

Xiangqian Kong [1,2,3], Zhihua Qiu [2,3,*], Lina Wu [2,3,4], Yunfei Lei [1] and Lisheng Chi [2,3,*]

[1] College of Chemistry and Materials Science, Fujian Normal University, Fuzhou 350000, China; xq20kong@fjirsm.ac.cn (X.K.); 107062019105@student.fjnu.edu.cn (Y.L.)
[2] Fujian Science and Technology Innovation Laboratory for Optoelectronic Information of China, Fuzhou 350116, China; nl20wu@fjirsm.ac.cn
[3] Fujian Key Laboratory of Fuel and Materials in Clean Nuclear Energy System, Fujian Institute of Research on the Structure of Matter, Chinese Academy of Sciences, Fuzhou 350002, China
[4] College of Chemistry, Fuzhou University, Fuzhou 350000, China
* Correspondence: qzh@fjirsm.ac.cn (Z.Q.); lchi@fjirsm.ac.cn (L.C.)

Table of contents

Figure S1. XRD results diagram of $Ca_2Ga_2(Ge_{1-x}Si_x)O_7$:y%$Eu^{2+.}$

Figure S2. Excitation and emission spectra of $Ca_2Ga_2(Ge_{1-x}Si_x)O_7$:1%$Eu^{2+.}$

Figure S3. Data point plot of ln(I_0/I) vs 1/kT ($Ca_2Ga_2(Ge_{0.5}Si_{0.5})O_7$:1.0%$Eu^{2+}$).

Figure S4. Normalized intensity of $Ca_2Ga_2(Ge_{0.5}Si_{0.5})O_7$:1%$Eu^{2+}$ vs Temperature.

Figure S5. SEM images at different scales of $Ca_2Ga_2(Ge_{0.5}Si_{0.5})O_7$:1.0%$Eu^{2+.}$

1. Figures

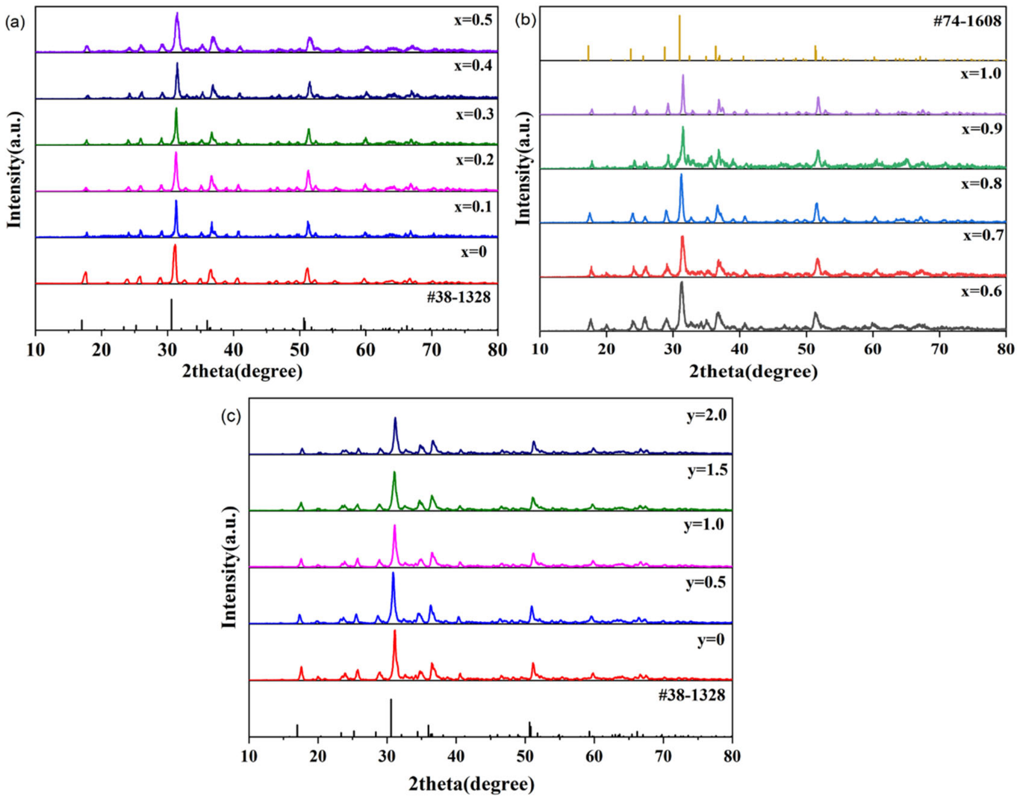

Figure S1. XRD results diagram of $Ca_2Ga_2(Ge_{1-x}Si_x)O_7$:y%$Eu^{2+}$.(**a**) x = 0-0.5, y = 1 (**b**) x = 0.6-1, y = 1 (**c**) x = 0.5, y = 0-2.

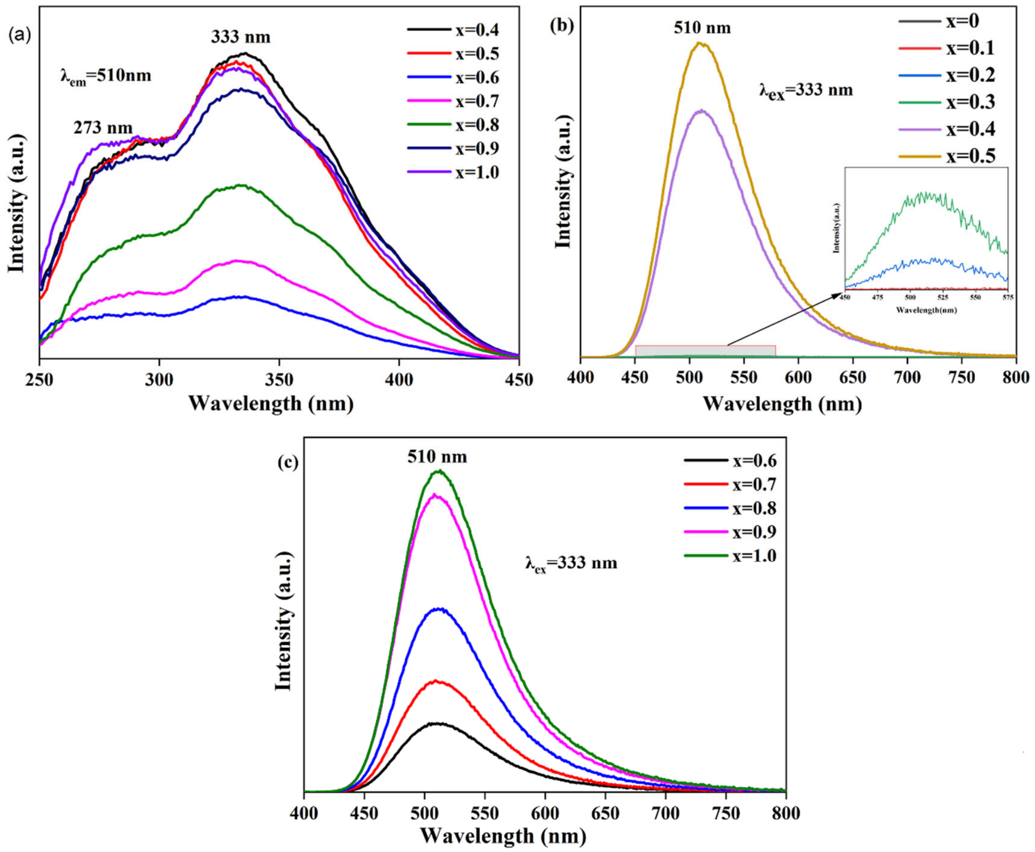

Figure S2. Excitation and emission spectra of $Ca_2Ga_2(Ge_{1-x}Si_x)O_7$:1%$Eu^{2+}$. (**a**) Excitation spectra, x = 0.4-1. (**b**) Emission spectra, x = 0-0.5. (**c**) Emission spectra, x = 0.6-1.

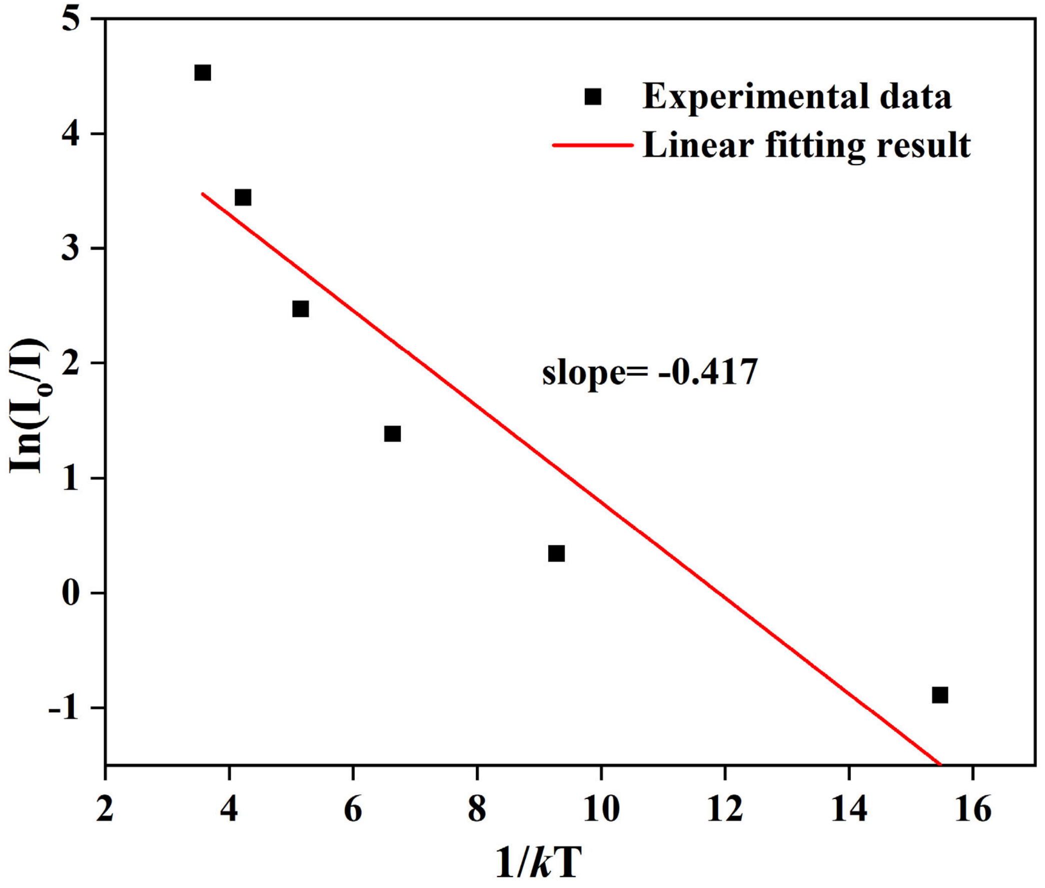

Figure S3. Data point plot of ln(I_0/I) vs 1/kT ($Ca_2Ga_2(Ge_{0.5}Si_{0.5})O_7$:1.0%$Eu^{2+}$).

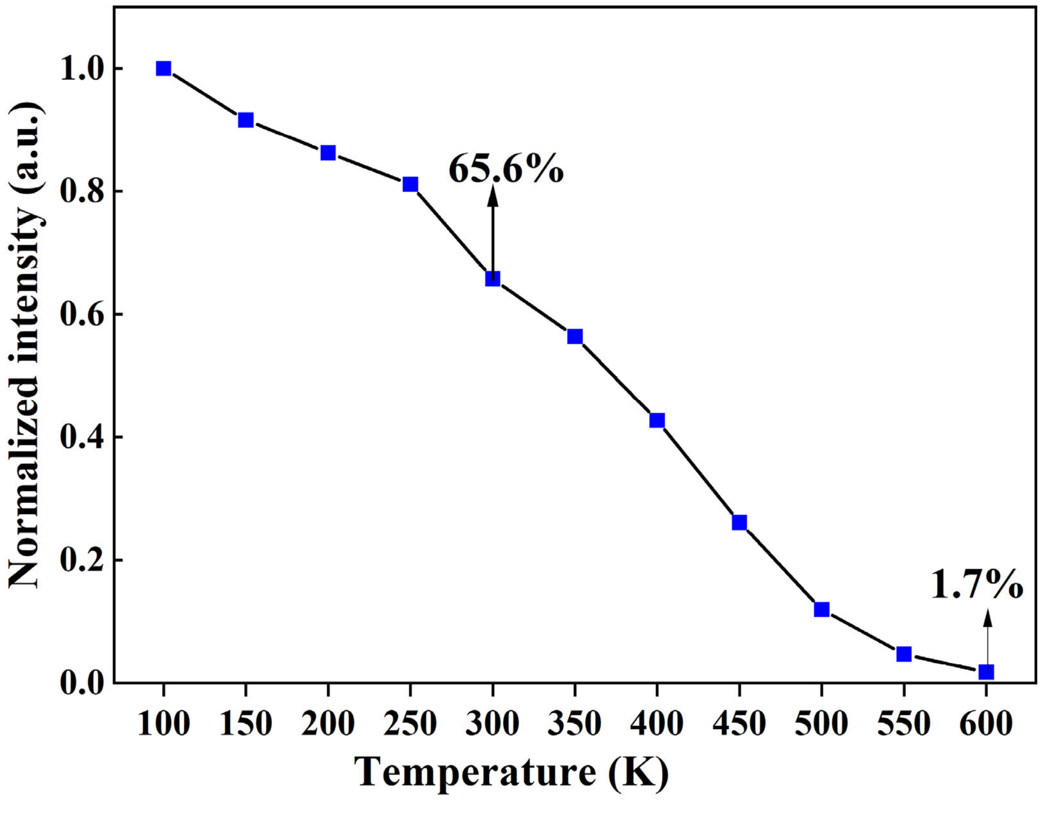

Figure S4. Normalized intensity of $Ca_2Ga_2(Ge_{0.5}Si_{0.5})O_7$:1%$Eu^{2+}$ vs Temperature.

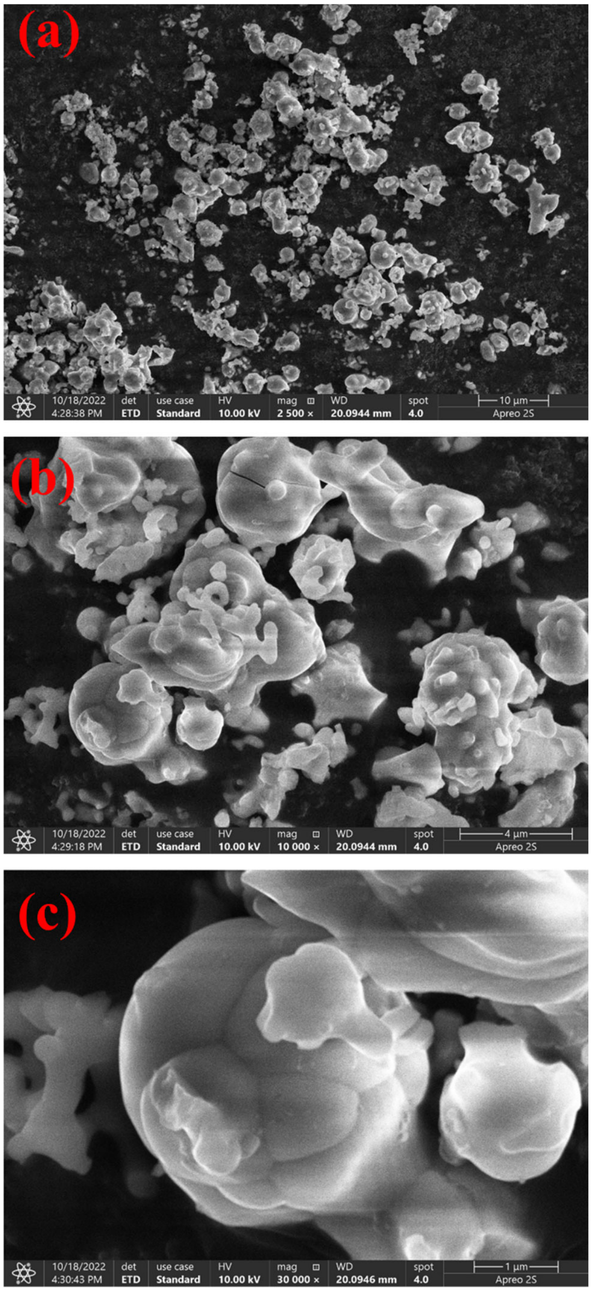

Figure S5. SEM images at different scales of $Ca_2Ga_2(Ge_{0.5}Si_{0.5})O_7$:1.0%$Eu^{2+}$. (**a**) 10 μm (**b**) 4 μm (**c**) 1 μm.
